# Advances in pediatric acute kidney injury pathobiology: a report from the 26th Acute Disease Quality Initiative (ADQI) conference

**DOI:** 10.1007/s00467-023-06154-y

**Published:** 2023-10-04

**Authors:** Michelle C. Starr, Erin Barreto, Jennifer Charlton, Molly Vega, Patrick D. Brophy, O. N. Ray Bignall, Scott M. Sutherland, Shina Menon, Prasad Devarajan, Ayse Akcan Arikan, Rajit Basu, Stuart Goldstein, Danielle E. Soranno, the ADQI 26 workgroup

**Affiliations:** 1 Department of Pediatrics, Division of Nephrology, Indiana University School of Medicine, Riley Hospital for Children, 1044 W. Walnut Street, Indianapolis, IN 46202 USA; 2 Pediatric and Adolescent Comparative Effectiveness Research, Department of Pediatrics, Indiana University School of Medicine, Indianapolis, IN USA; 3 Department of Pharmacy, Mayo Clinic, Rochester, MN USA; 4 Department of Pediatrics, Division of Nephrology, University of Virginia, Charlottesville, VA USA; 5 Renal and Apheresis Services, Texas Children’s Hospital, Houston, TX USA; 6 Department of Pediatrics, Golisano Children’s Hospital, University of Rochester, Rochester, NY USA; 7 Department of Pediatrics, Division of Nephrology and Hypertension, Nationwide Children’s Hospital and The Ohio State University College of Medicine, Columbus, OH USA; 8 Department of Pediatrics, Division of Nephrology, Stanford University School of Medicine, Stanford, CA USA; 9 Division of Pediatric Nephrology, Seattle Children’s Hospital and University of Washington, Seattle, WA USA; 10 Department of Pediatrics, Division of Nephrology and Hypertension, Cincinnati Children’s Hospital Medical Center, University of Cincinnati, Cincinnati, OH USA; 11 Department of Pediatrics, Divisions of Critical Care and Nephrology, Baylor College of Medicine, Texas Children’s Hospital, Houston, TX USA; 12 Department of Pediatrics, Division of Critical Care, Northwestern University, Chicago, IL USA; 13 Department of Bioengineering, Purdue University, West Lafayette, IN USA

**Keywords:** Acute kidney injury, Animal models, Translational research, Outcomes, Pediatrics, Neonates, Development as a biological variable

## Abstract

**Background:**

In the past decade, there have been substantial advances in our understanding of the pathobiology of pediatric acute kidney injury (AKI). In particular, animal models and studies focused on the relationship between kidney development, nephron number, and kidney health have identified a number of heterogeneous pathophysiologies underlying AKI. Despite this progress, gaps remain in our understanding of the pathobiology of pediatric AKI.

**Methods:**

During the 26th Acute Disease Quality Initiative (ADQI) Consensus conference, a multidisciplinary group of experts discussed the evidence and used a modified Delphi process to achieve consensus on recommendations for opportunities to advance translational research in pediatric AKI. The current state of research understanding as well as gaps and opportunities for advancement in research was discussed, and recommendations were summarized.

**Results:**

Consensus was reached that to improve translational pediatric AKI advancements, diverse teams spanning pre-clinical to epidemiological scientists must work in concert together and that results must be shared with the community we serve with patient involvement. Public and private research support and meaningful partnerships with adult research efforts are required. Particular focus is warranted to investigate the pediatric nuances of AKI, including the effect of development as a biological variable on AKI incidence, severity, and outcomes.

**Conclusions:**

Although AKI is common and associated with significant morbidity, the biologic basis of the disease spectrum throughout varying nephron developmental stages remains poorly understood. An incomplete understanding of factors contributing to kidney health, the diverse pathobiologies underlying AKI in children, and the historically siloed approach to research limit advances in the field. The recommendations outlined herein identify gaps and outline a strategic approach to advance the field of pediatric AKI via multidisciplinary translational research.

## Introduction

Over the past decade, significant advances spanning the research continuum have expanded our understanding of pediatric acute kidney injury (AKI). Basic science work explains kidney development and the diverse mechanisms of AKI, translational research extends these findings into clinical medicine, epidemiologic studies characterize the burden and clinical impact of the disease, and community interventions link these innovations to patients and their families.

Despite these advances, due to a number of challenges, there remain significant gaps in our understanding of the pathobiology of pediatric AKI. The diversity of “pediatric” patients ranges from premature neonates to adult-sized adolescents. A siloed approach to research and discovery in the field leads to a disconnect between bench, translational, and clinical research. Structural challenges exist in pediatric AKI research, including limited dedicated research funding and a lack of inclusion of pediatric or birth information in adult kidney studies.

The full ramifications of pediatric AKI remain unknown. The acknowledgment that AKI predisposes patients to developing chronic kidney disease (CKD) means that pediatric AKI survivors will become adults with CKD [1]. Beyond the kidney-specific outcomes of pediatric AKI across the life course, even less is known about the systemic sequelae that pediatric AKI may have on normal growth, development, and other organ dysfunction.

To address the need to improve research and clinical care in pediatric AKI, the 26th Acute Disease Quality Initiative (ADQI) conference was convened. We address three main questions from the conference in this article:

1. What are the biopsychosocial factors that lead to optimal kidney development and to a healthy kidney lifespan?
2. What are the biopsychosocial factors that lead to deviations from an optimal kidney life course?
3. What are the necessary components to create an integrated framework for translational research to mitigate pediatric AKI and optimize lifelong kidney outcomes?

## Methods

The 26th ADQI Consensus conference, the first ADQI devoted to pediatric AKI, was held over 3 days in Napa, CA, in November 2021, and included an interdisciplinary group of clinicians and researchers from North and South America, Africa, Asia, and Europe. Relevant disciplines were well represented, including pediatric nephrology, pediatric and adult critical care, pharmacy, epidemiology, health services research, advocates, pediatric nephrology nurses, nutritionists, and patients. As previously described, this consensus meeting followed the established ADQI process, with the broad objective to provide expert-consensus statements via interpretation of current knowledge for use by clinicians according to professional judgment and to identify evidence gaps to establish research priorities [2].

Workgroup 5 sought to develop consensus statements to improve future translational research in pediatric AKI and an understanding of the factors that lead to optimal kidney health and deviations from this optimal state. Given the large area of focus of workgroup 5 (pathobiology, pharmacology, and nutrition) and the task of answering 5 key questions, the 2 questions involving pharmacology and nutrition are addressed in a separate manuscript. The consensus-building process, informed by objective review of articles by workgroup members, used a modified Delphi method based on evidence when possible, with the ultimate goal of addressing the 3 key questions and articulating a research agenda to address existing knowledge gaps [3]. Consensus statements required two-thirds majority vote of all ADQI participants. Herein, we provide a summary of the current knowledge of factors impacting optimal kidney development and a healthy kidney lifespan, and more detailed recommendations regarding research approaches to be used as a framework for the advancement of pediatric AKI care.

## Results

A critical recommendation of the consensus panel was a need to improve pediatric AKI research across the continuum from bench to bedside, as follows:

*Successful pediatric translational AKI research programs include diverse teams using reverse translational approaches in partnership with clinical and epidemiological findings that prioritize development as a biologic variable. Sufficient support including pediatric specific government and industry funding along with meaningful partnerships among health professionals is necessary to understand and leverage the unique aspects of pediatric AKI to address kidney health and disease across the life course.*

In order to fully address the current state of evidence leading this consensus recommendation and opportunities for future translational research advancement, we sought to answer the 3 questions related to pathobiology developed during the pADQI conference.

### Question 1. What are the biopsychosocial factors that lead to optimal kidney development and to a healthy kidney lifespan?

Pediatric kidney disease research, and specifically pediatric AKI, is uniquely challenged due to the wide spectrum of progressive developmental states included within pediatric medicine. For example, a premature neonate and a post-pubertal teenager are both considered pediatric patients despite differences in development, nephron number, risk for, and impact of episodes of AKI on future health. In order to better understand the spectrum of pediatric AKI and to advance our understanding of the diverse pathobiology of this condition, we need to better understand development as a biologic variable (DABV) and the factors which contribute to maximum nephron number, kidney development, and kidney health throughout the lifespan. Nephrogenesis is complete at approximately 34 weeks gestation and kidney function continues to develop and mature throughout the first 2 years of life. Glomerular and tubular function undergo constant change and maturation throughout this period. Herein, we use DABV to assess the potential effect this flux in kidney development has on AKI incidence, severity, and outcomes (Table 1).

**Table 1:** Gaps in understanding of biopsychosocial factors leading to deviations from optimal kidney life course path

Area	Gap	Potential opportunities
Development as a biologic variable	Wide spectrum of progressive developmental states included within pediatric medicine	1. Pediatric and neonatal specific animal models 2. Pre-clinical studies using models that incorporate kidney development 3. Use of stem cells and organoids to better understand the role of development
Factors influencing nephron number	Wide variability in human nephron number Poor understanding of maternal, perinatal and neonatal factors influencing nephron number and subsequent development Poor understanding of nephron number decrease over time	1. Determination of what inhibits or drives ideal nephrogenesis 2. Specific mechanisms that influence nephrogenesis and final nephron number 3. Role of maternal malnutrition and food insecurity in nephron number and recovery from AKI 4. Neonatal and pediatric specific animal models to investigate the impact of AKI on long-term kidney function 5. Larger and more inclusive studies to understand impact of nephron development on age-related kidney function decline
Role of AKI in kidney dysfunction	Evidence of progression from AKI to CKD is not well established in pediatric patients	1. Larger studies including consistent AKI and CKD definitions are needed in order to define the absolute risk of CKD development 2. Importance of timing of pediatric AKI episode on kidney health
Sex as a biologic variable	Few clinical studies capture pubertal stage or measure sex hormones	1. Using models which take into account SABV incorporating DABV could be applied to inform translational studies in a diverse population of patients along the gender spectrum 2. Clinical and translational studies which incorporate pubertal stage and measure sex hormones
Systemic and long-term outcomes after AKI	Most pre-clinical AKI research has been confined to relatively short-term outcomes	1. Longer term studies in pre-clinical models specific to pediatric AKI 2. Use of larger clinical populations or registry-based studies to better understand the systemic role of AKI

#### Current understanding of development as a biological variable

Kidney development and nephron number is a result of a complex interplay between distinct embryologically derived cell populations [4]. Many factors that appear to impact DABV, including the genetic and molecular regulation of kidney development and the mechanisms of kidney development, have been extrapolated from animal work [5, 6]. Additionally, the role of epigenetic programming in premature birth remains unclear. Recent animal studies have shown that perinatal epigenetic programming through alterations in the kidney corticosteroid signaling pathways may contribute to DABV following premature birth [7]. In addition, there is now compelling pre-clinical evidence for the induction of an embryonic phenotype in injured tubule cells of the adult kidney, with robust re-expression of genes normally present only in the developing kidney [8]. This switch to the embryonic state is likely critical for regeneration of tubule cells lost during AKI. The identified developmental genes and gene products that accelerate repair in the adult kidney represent novel future therapeutic targets.

#### Factors impacting nephron number

Factors impacting nephron number and development impact kidney health along the entire life course, and are therefore an issue of major consequence for pediatric AKI and long-term outcomes. There is wide variability in human nephron number as early as the neonatal period due to DABV [9]. In humans, the completion of nephrogenesis coincides with the completion of gestation; nephron number is therefore impacted by gestational age at birth [9]. The cessation of human nephrogenesis is relatively consistent across studies and has been reported to occur between 32 and 36 weeks’ gestation. However, kidney function continues to develop and mature throughout the first 2 years of life. Nephrogenesis was recently documented at 37 weeks’ gestation, and several adult studies have found a correlation between birth weight, glomerular number, and risk of CKD later in life [10, 11]. It is not currently possible to determine when nephrogenesis is complete in humans because the data regarding the window of nephrogenesis is derived exclusively from post-mortem studies [11]. The duration of human nephrogenesis is likely variable and may be a factor in an individual’s nephron endowment.

From an evolutionary perspective, fetal response to intrauterine stress is to reduce somatic growth and the growth of any organ not vital to early survival [12]. For the kidney, a “surplus” of nephrons is not likely to provide an early survival benefit. However, the trade-off for a lower initial nephron number is the increased risk for CKD as a person ages [13]. Beyond the many genes that confer a low nephron number and result in congenital anomalies of the kidney and urinary tract, low birth weight has been recognized as a risk factor for the development of CKD [14]. However, the specific mechanisms that influence nephrogenesis and final nephron number, both in utero and ex utero, and the processes regulating nephron loss are not well understood.

Any factor that mitigates optimal baseline kidney health likely predisposes patients to developing AKI and may also impair recovery. Hence, these factors, when perturbed early in life, may have a significant impact on kidney health across the lifespan.

### Question 2. What are the biopsychosocial factors that lead to deviations from an optimal kidney life course path?

#### Biopyschosocial factors impacting nephron development

Many other factors influence nephron number in utero and ex utero [9]. Maternal protein restriction and deficiency of iron or vitamin A can reduce nephron endowment [15–17]. In animal models, periods of maternal fasting are associated with a reduction in nephron number in offspring [18], and this appears to be mediated in part by an associated congenital nephron deficit occurring from intrauterine growth restriction [19]. Reduced glomerular filtration rate and albuminuria accompany nephron reduction with numerous studies demonstrating an increased prevalence of microalbuminuria and proteinuria among adults born low birth weight [20–22]. It is challenging to separate birth weight and gestational age from low birth weight (LBW), which is often used as a surrogate marker of prematurity [23]. In epidemiologic studies, food insecurity, a social determinant of health associated with malnutrition, was associated with higher rates of CKD and faster progression to kidney failure [24, 25]. More studies are needed to assess the role of maternal malnutrition and food insecurity in nephron number and recovery from AKI in children.

Over time, nephron number appears to naturally decrease in humans. In the last decade, investigators have capitalized on the unique setting of living donor kidney transplantation to study nephron number, finding an estimated average glomerular number in healthy donors of nearly 900,000, and have shown a lower glomerular number in kidney donors of older ages [26]. These data are limited by the mostly cross-sectional nature of epidemiologic studies. The potential impact of nephron development during infancy and early childhood on age-related kidney function decline remains unknown.

#### Biopsychosocial contributions to nephron number

In the 1980s, Brenner proposed that low nephron endowment would lead to impaired sodium excretion, glomerular hypertrophy, and glomerular hypertension, leading to the subsequent development of glomerulosclerosis and further decline of nephron number [27]. This hypothesis has not been tested in vivo. In preclinical model of LBW, those born LBW had a greater proportional increase in kidney size and glomerular hypertrophy compared to normal birth weight controls [28]. In settings of reduced nephron number, this compensation may be exaggerated and could lead to accelerated loss of kidney function [29, 30]. There is still a great deal to understand regarding the thresholds of nephron number to cause hypertrophy and the capacity and limit of glomerular hypertrophy.

Greater severity or more rapid progression of kidney disease has been shown among adults born with LBW and/or prematurity [27]. The incidence of adult kidney failure is 40% greater among those with birth weights under 2.5 kg compared to those with normal birth weight [31]. Children born LBW had lower glomerular density with glomerular enlargement on kidney biopsy compared to those born at normal birth weight [32]. Focal segmental glomerulosclerosis (FSGS) is the predominant histologic finding in kidney biopsies of adolescents and adults born preterm [33]. In children with FSGS on biopsy, those with LBW had hyperplastic glomeruli with fewer podocytes and more sclerotic lesions [16, 34]. Taken together, these findings suggest the concept of a “podocytopathy” from preterm birth [11, 12, 33, 35] or growth restriction, which may contribute to the variety of outcomes seen in patients with nephrotic syndrome, FSGS, or IgA. Biopsychosocial variables impact the risk of preterm birth, and these same variables may continue to impact kidney development after birth.

#### AKI in development of kidney dysfunction

The risk of CKD or kidney failure after AKI has been well detailed in adult studies [36]. In 2019, an updated meta-analysis and systematic review identified 82 studies including over 2 million adult patients who had AKI [37]. Following an episode of AKI, adults had a hazard ratio (HR) of 2.67 for new or progressive CKD (CI 1.99–3.58), 4.81 for kidney failure, and 1.8 for death.

Researchers have proposed that AKI and CKD are interconnected syndromes, and not separate disease processes [38]. The mechanisms for progression to CKD are incompletely understood, but likely are secondary to maladaptive repair, ongoing inflammation, and disordered regeneration [39]. The evidence of progression to CKD from AKI is less established in pediatric patients. In a systematic review of 346 patients with a mean follow-up of 6.5 years, the cumulative incidence of abnormal GFR < 90 mL/min/1.73 m^2^ was 6% [40]. Similarly, 10% of pediatric patients had CKD (defined as albuminuria and/or GFR < 60) 1 to 3 years following AKI, while 47% were at risk for CKD (defined as GFR < 90, hypertension or GFR > 150) [41, 42]. In 100 pediatric patients with nephrotoxin-associated AKI, 70% had evidence of residual kidney damage 6 months after AKI [43]. These studies suggest that the impact an episode of early childhood AKI has on long-term kidney health is likely different than the impact of AKI on adults with fully developed kidney function. These impacts likely change significantly with respect to kidney DABV. Larger pediatric studies that include consistent AKI and CKD definitions are needed in order to define the absolute risk of CKD development after childhood AKI.

A thorough review of translational AKI models has recently been published [44]. These models provide a robust platform to investigate the various pathophysiologies of AKI (i.e., sepsis, ischemia–reperfusion, nephrotoxic). However, most pre-clinical models do not account for the additional complexity of pediatric AKI imparted by DABV. Furthermore, the effect of an episode of AKI during various stages of kidney development remains unclear. We propose that the severity, duration, and timing of an AKI episode with respect to DABV impact the long-term kidney and global health outcomes across the child’s lifespan. DABV can be investigated by using models that incorporate kidney development. For example, Chevalier et al. developed a partial-reversible unilateral ureteral obstruction model in rat pups that enables the investigation of clinically relevant partial obstruction in neonates [45]. Liberio et al. recently published new models of pediatric ischemia–reperfusion and nephrotoxic AKI in rat pups and demonstrated the kidney-lung crosstalk in pulmonary vascularization [46]. Stem cells and organoids can be investigated using reverse-translational approaches spanning the pathway of nephron and organoid development [47]. Such models can be used to probe the unique aspects of pediatric AKI compared to AKI in adults.

#### Sex as a biologic variable

Sex is an important biologic variable in the development of AKI, and progression to CKD [48–52]. The NIH has made a call to action for the inclusion of both sexes in pre-clinical and clinical research [53]. Established sex differences in AKI (e.g., female sex is protective in ischemia–reperfusion AKI and deleterious in some forms of nephrotoxin-mediated AKI) have stymied the inclusion of both males and females in pre-clinical animal studies. Determining the mechanism of protective sex biases would allow researchers to identify novel therapeutic targets that benefit both sexes.

In pediatrics, sex as a biological variable (SABV) is further confounded by DABV. Hormone levels change significantly from pre-pubertal to peri-pubertal to post-pubertal stages, and few clinical studies capture pubertal stage or measure sex hormones. Complexity is further added for the care of intersex and transgendered youths undergoing sex affirming hormonal therapy. Pre-clinical animal models exist which unconfound the effects of sex hormones (estrogen and testosterone) from sex chromosomes (XX, XY) [44]. Using models which take into account SABV incorporating DABV could be applied to inform translational studies in a diverse population of patients along the gender spectrum.

#### Investigating systemic and long-term outcomes after AKI

Most pre-clinical AKI research has been confined to relatively short-term outcomes. Additionally, there is a growing appreciation that AKI results in systemic sequelae [54]. Studies have shown that premature infants with AKI have worse short- and long-term pulmonary outcomes and neurologic outcomes [55, 56]. Recent preclinical studies demonstrate long-term cardiovascular and growth effects after ischemia–reperfusion AKI [37, 57, 58]. Septic AKI is associated with worse functional outcomes; this same observation has been demonstrated in survivors of continuous kidney replacement therapy [59–61]. The potential effects of AKI with respect to DABV on these long-term outcomes are unknown and may have significant implications for global health outcomes of children who suffer an episode of AKI. Preclinical models specific to pediatrics are needed to bridge these knowledge gaps.

### Question 3: What are the necessary components to create an integrated framework for translational research to mitigate pediatric AKI and optimize lifelong outcomes?

#### An integrated approach to translational research

AKI is a syndrome with heterogeneous causes and multiple clinical phenotypes, which require a detailed understanding of DABV. Traditionally, translational AKI research has used numerous pre-clinical models to investigate the mechanisms of AKI pathophysiology and outcomes with a goal to bring potential therapies to the bedside [44]. Until recently, modeling of AKI was primarily based on animal and cell culture models. Numerous animal models of AKI have been established, each with translational strengths, advantages, and challenges. However, these models of AKI differ significantly from human AKI in molecular and cellular responses, biomarkers, and clinical manifestations and transferring animal and pre-clinical data from animal models to humans is inherently challenging [62, 63]. In addition to the limitations of translating animal models to human research, translating these animal models for pediatric AKI faces additional challenges: (1) the vast majority of preclinical AKI models evaluate short-term outcomes, and (2) these models are performed in young adult animals. As a result, the standard preclinical AKI model is unable to recapitulate the unique aspects of growth and development in pediatric medicine and also fails to capture the important aspect of the long-term systemic and kidney sequalae of AKI along the life course.

The past two decades have seen a paradigm shift, moving towards personalized human-based models to study human disease. Novel in vitro systems for AKI diagnosis include human-induced pluripotent kidney stem cells [64–66], human stem cell-derived kidney organoids [67, 68], and human kidney tumor-derived stem cells [69]. Preclinical models of AKI have resulted in significant advances in the pathophysiology of AKI and identified several non-dialytic therapeutic targets which include the following [70, 71]: anti-inflammatory [72, 73], anti-necrosis/apoptosis [74, 75], antioxidants [76], anti-sepsis [77], growth factors [78, 79], and vasodilators [80, 81]. Additionally, prevention of AKI has been demonstrated with methylxanthines [82, 83]; in particular, caffeine administration may reduce the risk of AKI in premature neonates [84]. More novel therapeutics such as mesenchymal stem cells (MSCs) have also been shown to improve outcomes in AKI [85, 86]. Numerous injectable hydrogel systems have been studied for local delivery of therapeutics to the kidney [87–93]. These injectable systems allow for sustained local delivery over a set period of time [94]. Several potential therapeutics are currently undergoing clinical trials [95].

Despite these advances, there have been challenges translating therapies into clinical use, and many promising preclinical therapies have failed to demonstrate efficacy in human trials [96]. The NIDDK published guidelines to overcome these barriers, and recommends utilizing a reverse-translational approach whenever possible [97]. In this manner, the preclinical models are designed to match the clinical intervention with respect to disease pathophysiology, timing of intervention, and outcome measures. The group also recommended incorporation of serum and urine biomarkers in addition to functional assessments of kidney function to better translate the spectrum of kidney injury between preclinical and clinical studies [97].

To improve the translational impact of these research efforts, we advocate for an approach that integrates pre-clinical, clinical, and epidemiological research efforts so that they can inform one another in study design and outcome measures and that pediatric-specific pre-clinical models are employed whenever available (Table 2).

**Table 2:** Consensus recommendations of potential components to create an integrated framework for translational research

Creating and promoting an integrated approach to translational research	1. Information sharing is an integral element of basic scientists to advance their findings and communicate with clinical research investigations 2. In an ideal state, every pre-clinical study would include a clinical research colleague (and vice versa). For this to be practical, it should be supported financially and encouraged by collaborative networks. This would help de-silo our field further and foster collaboration 3. The translatability of preclinical models is strengthened when they match the clinical intervention with respect to disease pathophysiology, timing of intervention, and outcome measures 4. Preclinical studies are strengthened when they incorporate the use of serum and urine biomarkers in addition to functional assessments of kidney function 5. Research teams must identify core outcomes based on mutual goals and priorities that are meaningful for both patients and healthcare teams 6. Research teams must successfully disseminate their findings and engage key stakeholders in order to improve care and serve the community. This includes sharing new knowledge intentionally and in co-equal partnership with providers, patients, families, and the community. Tailored dissemination can enhance awareness and provision of healthcare by providers, empower patients and families, spur government to improve policy and funding, and engage communities with a focus on equity 7. Collaborations among research teams, educators, and community representatives may enable innovative approaches to reach the community they serve
Increasing innovation through diversity	1. Institutional and system-wide support is needed to recruit, train, support, retain, and amplify a robust pipeline of translational researchers from diverse backgrounds 2. Development and promotion of diverse teams improves innovation and facilitates success throughout all stages of research 3. Programs that specifically incentivize the building of diverse teams, both at local levels and those which are tied to funding (e.g., NIH), will advance the field of pediatric translational research
Adequate funding and investment	1. Specific pediatric funding in kidney focused research studies through NIDDK and other NIH institutions will advance the field of pediatric AKI research and kidney health along the life course 2. Inclusion of kidney-specific outcomes in pediatric studies will de-silo the field of nephrology from overall pediatric health 3. Inclusion of children in clinical investigations should be prioritized in disease processes shared between pediatric and adult patients 4. Collaboration among researchers in various specialties optimizes the translatability of research investigations

#### Diversity increases innovation

Diverse teams improve innovation in research and clinical delivery of pediatric AKI care. Beyond the diversity of expertise required to inform and perform translational AKI research, teams are further strengthened by the diversity of their background, including but not limited to, sex, gender, age, race, ethnicity, and ability [98]. Empirical data demonstrate that diverse research teams produce more innovative research; yet, publications from authors who identify as under-represented in medicine are less likely to be highly cited [98, 99].

Research investigations are strengthened by the inclusion of team members with a broad array of expertise, such as the inclusion of clinical researchers on pre-clinical work and health service researchers, patients and patient advocates, and implementation scientists to understand how to optimally deploy clinical and research findings [44]. A truly integrated research approach includes not only investigations throughout the basic science to epidemiologic spectrum, but also incorporates digital health tools and community partnerships to better meet patients and families in their communities and make them partners in health [100]. Health service and epidemiological studies may benefit from inclusion of specific outcome measures guided by pre-clinical work. Conversely, epidemiological findings can drive pre-clinical mechanistic investigations via reverse-translational approaches [63]. In addition, partnerships between patients, patient advocates, clinicians, researchers, industry, and policy makers can help identify core outcomes. Such outcomes developed on shared priorities have the potential to make research more meaningful for the patients, and the clinicians taking care of them [101].

**Fig. 1:**
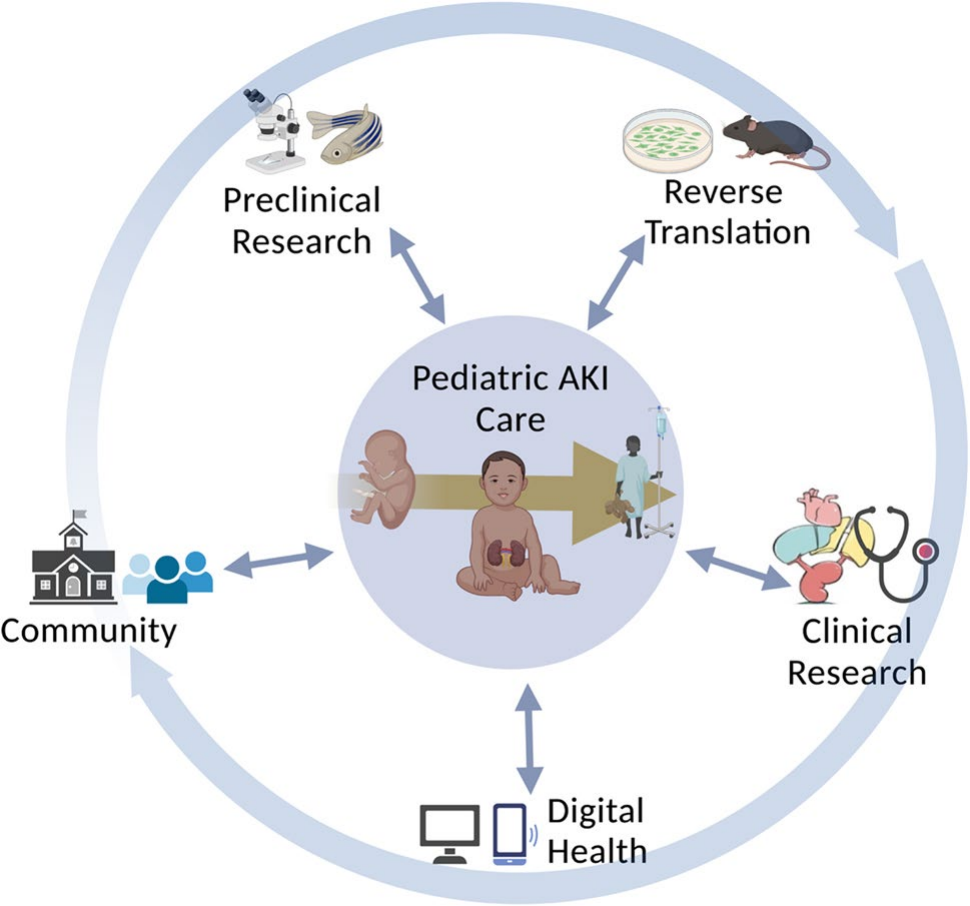
Visual representation of an integrated framework for translational research, including the multiple silos of research including preclinical research, reverse translation approaches, and clinical research. Along with community engagement and digital health, these elements all interact in a multi-directional collaborative approach to impact pediatric AKI care

Beyond the research setting, research teams must successfully disseminate their findings and engage key stakeholders in order to improve care and serve the community. This includes sharing new knowledge in context-appropriate ways with members of the healthcare team, patients, families, and the community [3]. This need is discussed in further depth by Workgroup 6. Collaborations among research teams, educators and community representatives may enable innovative approaches to reach the communities they benefit (Fig. 1). Engagement of government and funding agencies in such efforts can increase awareness, and potentially improve resources available to healthcare teams [102].

To bolster diversity and innovation in pediatric AKI research, individual, institutional, and system-wide support is needed to recruit, train, support, retain, and amplify a robust pipeline of translational researchers from diverse backgrounds [103]. Diversity in the makeup of research and clinical teams should be deliberately supported, encouraged, and rewarded by research enterprises and institutions, with specific attention paid to efforts of equity and inclusion. The Society for Pediatric Research recently published a Call to Action with specific and meaningful ways academic organizations, schools, departments, and faculty members can improve the diversity of the pediatric scientific workforce [104].

#### Adequate funding and investment

In order to advance our understanding of pediatric AKI and identify therapeutic targets to optimize outcomes throughout the life course, robust, sustained, and predictable funding support is required. As a whole, kidney disease research is underfunded, with an NIH investment of < 1% of the cost of kidney care [105]. This inequity in funding is likely even more stark in pediatric kidney disease research. However, it is not clear how much support pediatric kidney disease research currently receives, because this has not been tracked by the NIH.

The NIDDK has recently announced its 5-year strategic plan to augment kidney disease research, which does not specifically prioritize or target pediatric kidney disease research. Beyond the USA, more global investment is warranted. Currently, the global action plan for the prevention and control of noncommunicable diseases does not include kidney disease; we join others in advocating for its inclusion [106]. In addition to research funding from governments and institutions, public–private partnerships and industry involvement should be encouraged. Appropriately designed regulations and incentives can foster investment into pediatric research by biopharmaceutical companies.

Pediatric kidney research should be prioritized given its impact on patients throughout their life course. Additionally, as developmental pathways are reactivated during and after AKI, a better understanding of these pathways is essential for better understanding of recovery from AKI versus progression to CKD. In light of the burden of AKI and CKD in the adult population, investments in understanding these developmental pathways may be an efficient use of limited research funding. Kidney-related outcomes should be a focus of not only NIDDK studies but should be included in other pediatric studies and clinical trials, including targeted support from *Eunice Kennedy Shriver* National Institute of Child Health and Human Development (NICHD) [107]. During the early neonatal period and childhood is the only time where nephron number can be impacted, either optimizing this for the life course or setting up a child for a lifetime risk of decreased nephron number and increased risk of CKD. Additionally, as pediatric patients with AKI are at-risk to become adults with CKD, and as seriously ill children are future adult patients, aligning and combining research efforts in adult and pediatric nephrology would benefit both fields and improve outcomes for patients along the life course with kidney disease. Inclusion of children in clinical investigations and collaboration among researchers in various specialties optimizes the translatability of research investigations. For example, coordinated projects such as the Kidney Precision Medicine Project represent opportunities to include pediatric samples, and in doing so, broaden and bolster the research findings.

## Conclusion

Despite recent advances in our understanding of the pathobiology of pediatric AKI, there remain large gaps in our understanding of the diverse spectrum of pediatric AKI. Further research advancements in the field require that pediatric translational AKI research programs focus on the unique aspects of development as a biological variable, and the impact of kidney health and disease across the life course. These efforts merit and require substantial support. In order to accomplish these goals, research must include diverse and multidisciplinary teams, be supported by robust and predictable funding from government and industry and employ meaningful partnerships with multiple other medical disciplines.

## Data Availability

This is not applicable to this manuscript as no new data was created.
